# Prospective Coding by Spiking Neurons

**DOI:** 10.1371/journal.pcbi.1005003

**Published:** 2016-06-24

**Authors:** Johanni Brea, Alexisz Tamás Gaál, Robert Urbanczik, Walter Senn

**Affiliations:** 1 Department of Physiology, University of Bern, Bern, Switzerland; 2 School of Computer and Communication Sciences and School of Life Sciences, Brain-Mind Institute, Ecole Polytechnique Fédérale de Lausanne, Lausanne, Switzerland; 3 Courant Institute of Mathematical Sciences, New York University, New York, New York, United States of America; 4 Center for Cognition, Learning and Memory, University of Bern, Bern, Switzerland; UCL, UNITED KINGDOM

## Abstract

Animals learn to make predictions, such as associating the sound of a bell with upcoming feeding or predicting a movement that a motor command is eliciting. How predictions are realized on the neuronal level and what plasticity rule underlies their learning is not well understood. Here we propose a biologically plausible synaptic plasticity rule to learn predictions on a single neuron level on a timescale of seconds. The learning rule allows a spiking two-compartment neuron to match its current firing rate to its own expected future discounted firing rate. For instance, if an originally neutral event is repeatedly followed by an event that elevates the firing rate of a neuron, the originally neutral event will eventually also elevate the neuron’s firing rate. The plasticity rule is a form of spike timing dependent plasticity in which a presynaptic spike followed by a postsynaptic spike leads to potentiation. Even if the plasticity window has a width of 20 milliseconds, associations on the time scale of seconds can be learned. We illustrate prospective coding with three examples: learning to predict a time varying input, learning to predict the next stimulus in a delayed paired-associate task and learning with a recurrent network to reproduce a temporally compressed version of a sequence. We discuss the potential role of the learning mechanism in classical trace conditioning. In the special case that the signal to be predicted encodes reward, the neuron learns to predict the discounted future reward and learning is closely related to the temporal difference learning algorithm TD(*λ*).

## Author Summary

Sensory inputs are often predictable. Lightning is followed by thunder, a falling object causes noise when hitting the ground, our skin gets wet when we jump into the water. Humans learn regularities like these without effort. Learned predictions allow to cover the ears in anticipation of thunder or close the eyes just before an object hits the ground and breaks into pieces. What changes in the brain when new predictions are learned? In this article, we present a mathematical model and computer simulations of the idea that the activity of a single neuron represents expected future events. Such a prospective coding can be learned in a neuron that receives input from the memory trace of a first event (e.g. lightning) and also input from the second event (e.g. thunder). Synaptic input connections from the memory trace are potentiated such that the spiking activity ramps up towards the onset of the second event. This deviates from the classical Hebbian learning that merely associates two events that are coincident in time. Learning in our model associates a current event to future events.

## Introduction

Animals can learn to predict upcoming stimuli. In delayed paired-associate tasks, animals learn to respond to pairs of stimuli (e.g. images A1-B1 and A2-B2) separated by a delay. These tasks can be solved by either keeping a memory of the first stimulus (A1 or A2) during the delay period (retrospective coding) or anticipating the second stimulus (B1 or B2) during the delay period (prospective coding). Monkeys seem to use both coding schemes [1]. Recordings in the prefrontal cortex of monkeys performing a delayed paired-associate task revealed single neurons with decreasing firing rate in response to a specific first stimulus (A1 or A2) and other neurons with ramping activity in trials where a specific second stimulus (B1 or B2) is anticipated [1, 2]. Thus, the firing rate of a neuron may encode not only past and current events, but also prospective events.

Learning to anticipate a future stimulus can also be observed in classical trace conditioning, where a conditioned stimulus (CS, e.g. sound of a bell) is followed after a delay by an unconditioned stimulus US (e.g. a sausage) that causes a response R (e.g. salivation) [3, 4]. After several repetitions of this protocol, the conditioned stimulus CS can elicit response R already before the onset of the unconditioned stimulus US.

A common experimental finding in these examples is the slowly ramping neuronal activity prior to the predicted event. In an experiment where mice choose to lick left or right in response to a tactile cue, the neural activity in the anterior lateral motor cortex ramps up in the waiting period before the response [5]. This activity pattern implements prospective coding as it indicates whether the animal will lick left or right. Serotonergic neurons in the dorsal raphe nucleus of mice show an activity ramp in a delay period between a predictive odor cue and the availability of a sucrose reward [6]. In rats that navigate a maze towards the learned position of a chocolate milk reward, the activity of striatal neurons increases while the rat approaches the reward position [7, 8]. In visual delayed paired associate tasks in which monkeys are trained to select a specific choice object that is associated with a previously shown cue object, increasing activity in the delay period was measured for neurons in the prefrontal cortex [1, 9, 10] and in the inferior temporal cortex [2, 11].

It is unclear how prospective coding emerges. The cue and the associated predictable event are typically separated by an interval of some seconds. On the other hand, synaptic plasticity, that is presumably involved in learning new associations, typically requires presynaptic and postsynaptic activity to coincide in a much shorter interval. Some tens of milliseconds is, for example, the size of the ‘plasticity window’ in spike-timing dependent plasticity; no synaptic change occurs, if presynaptic and postsynaptic spike are separated by more than the size of this plasticity window [12, 13]. This mismatch between the behavioral and the neuronal timescales begs the question how a neuronal system can learn to make predictions more than a second ahead. There are also plasticity mechanisms that can correlate pre- and postsynaptic spiking events that are separated by seconds [14, 15]. Yet, assuming many simultaneously active afferents, it remains unclear how the behaviourally relevant pair of pre- and postsynaptic spikes can be selected out of hundreds behaviourally irrelevant pairs.

In normative models of synaptic plasticity, the shape of the causal part of the plasticity window matches the shape of the postsynaptic potential (PSP), if the objective is to reproduce precise spike timings [16–18]. However, if the objective is to reproduce future activity, this specific learning rule is insufficient. Yet, as we demonstrate in this article, the same plasticity rule with only a slightly wider window also allows for learning a prospective code. With this mechanism, it is possible to learn an activity ramp towards a specific event in time, or to learn predicting a time-varying signal or a sequence of activities well ahead in time. In a 2-compartment neuron model, this mechanism leads to the dendritic prediction of *future* somatic spiking. The mechanism stands in contrast to the work of Urbanczik & Senn, where the current somatic spiking is predicted [18]. Despite this fundamental difference, the plasticity rules only differ in the width of the potentiation part of the plasticity window.

## Results

### Schematic description of the learning mechanism

**Fig 1:**
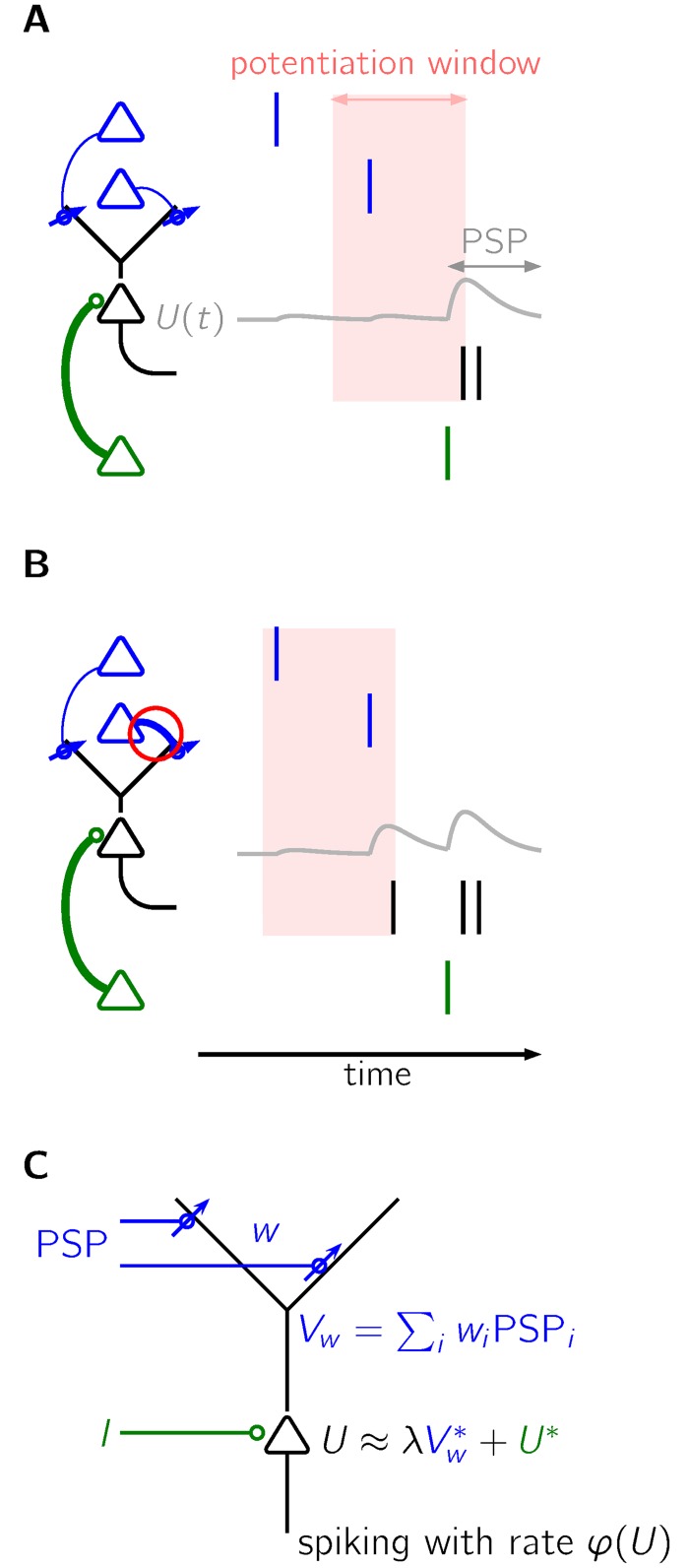
A learning mechanism that leads to prospective coding. **A** The signal to be predicted (target input) originates from the green neuron and depolarizes the black neuron (gray trace) such that it spikes (black lines). The synaptic connection between a blue neuron and the black neuron is strengthened if pre- and postsynaptic spikes lie within the red plasticity window of potentiation, which is slightly broader than a typical postsynaptic potential. **B** Due to the strengthened connection (red circle), the black neuron spikes already before the target input arrives. Since earlier presynaptic spikes now also lie within the potentiating plasticity window, the activity of the black neuron will be anticipated earlier, giving rise to prospective coding. **C** A spiking neuron receives input through plastic dendritic synapses with strengths *w*_*i*_ and an input *I* through static (i.e. non-plastic) synapses. The somatic membrane potential *U* is well approximated by the sum of attenuated dendritic input $V_{w}^{*}$ and attenuated somatic input U*.

Before defining the learning rule in detail, we provide an intuitive description. In a neuron with both static synapses (green connection in Fig 1A and 1B) and plastic synapses (blue in Fig 1A and 1B), we propose a learning mechanism for the plastic synapses that relies on two basic ingredients: spike-timing dependent synaptic potentiation and balancing synaptic depression. The synaptic connections are strengthened if a presynaptic spike is followed by a postsynaptic spike within a ‘plasticity window of potentiation’ (red in Fig 1A and 1B). The size of this plasticity window turns out to have a strong influence on the timing of spikes that are caused by strengthened dendritic synapses. If the plasticity window has the same shape as a postsynaptic potential (PSP), learned spikes are fired at roughly the same time as target spikes [16–18]. But if the plasticity window is slightly longer than the postsynaptic potential, learned spikes tend to be fired earlier than target spikes. More precisely, because of the slightly wider plasticity window of potentiation, presynaptic spikes may elicit postsynaptic spikes through newly strengthened connections (thick blue arrow in Fig 1B) even before the onset of the input through static synapses. These earlier postsynaptic spikes allow to strengthen the input of presynaptic neurons that spike even earlier. We refer to this as the bootstrapping effect of predicting the own predictions. As a result, a postsynaptic activity induced by the input through static synapses will be preceded by an activity ramp produced by appropriately tuned dendritic input. The neuron learns a prospective code that predicts an upcoming event.

### The 2-compartment neuron model

We consider a 2-compartment neuron model that captures important functional details of spiking neurons and is well suited for analytical analysis [18]. In this model (Fig 1C), a dendritic compartment receives input through plastic synapses with strength *w*. The voltage *U* of the somatic compartment is coupled to the dendritic voltage *V*_*w*_ and receives additional input *I* through static synapses,

$$C\;\dot{U}=-g_{L}U+g_{D}\left(V_{w}-U\right)+I\;,$$ (1)

where *g*_*L*_ is the leak conductance, *g*_*D*_ is the coupling conductance between soma and dendrite and *C*, the somatic capacitance. The dendritic potential *V*_*w*_ is given by a weighted sum of presynaptic inputs, i.e.

$$V_{w}\left(t\right)=\sum_{i}w_{i}{\text{PSP}}_{i}\left(t\right)=\sum_{i}w_{i}\sum_{t^{f}\in T_{i}}\kappa \left(t-t^{f}\right)$$ (2)

with plastic synaptic weights *w*_*i*_, postsynaptic potentials PSP_*i*_ that model the depolarization of the postsynaptic membrane potential due to the arrival of a presynaptic spikes at synapse *i*, set $T_{i}$ of spike arrival times at synapse *i* and spike response kernel *κ*. Spiking of the postsynaptic neuron is modeled as an inhomogeneous Poisson process with rate *φ*(*U*).

We model the input with time varying excitatory and inhibitory conductances *g*_*E*_ and *g*_*I*_ proximal to the soma such that

$$I\left(t\right)=g_{E}\left(t\right)\left(E_{E}-U\right)+g_{I}\left(t\right)\left(E_{I}-U\right)$$ (3)

as proposed by Urbanczik & Senn [18].

For large total conductance and slowly varying input, the somatic membrane potential *U*(*t*) is well approximated (see Methods) by its steady state solution

$$U\left(t\right)\approx \lambda \left(t\right)V_{w}^{*}\left(t\right)+U^{*}\left(t\right)\;,$$ (4)

where we introduced the attenuated dendritic potential

$$V_{w}^{*}\left(t\right)=\frac{g_{D}}{g_{L}+g_{D}}V_{w}\left(t\right)\;,$$ (5)

the attenuated somatic input

$$U^{*}\left(t\right)=\frac{g_{E}\left(t\right)E_{E}+g_{I}\left(t\right)E_{I}}{g_{\text{tot}}\left(t\right)}\;$$ (6)

and the ‘nudging’ factor

$$\lambda \left(t\right)=\frac{g_{L}+g_{D}}{g_{\text{tot}}\left(t\right)}\;,$$ (7)

with *g*_tot_(*t*) = *g*_*L*_ + *g*_*D*_ + *g*_*E*_(*t*) + *g*_*I*_(*t*), to be in accordance with Urbanczik & Senn [18]. The nudging factor *λ*(*t*) ∈ (0, 1] is close to 1 for small somatic input and equal to 1 if *g*_*E*_(*t*) + *g*_*I*_(*t*) = 0.

### Learning as dendritic prediction of the neuron’s future discounted firing rate

The plasticity rule we consider for the dendritic synapses can be seen as differential Hebbian in the sense that both the potentiation and depression term are a product of a post- and presynaptic term. The strength of synapse *i* is assumed to change continuously according to the dynamics

$${\dot{w}}_{i}=\eta \left(\alpha \;\varphi \left(U\right){\tilde{\text{PSP}}}_{i}-\varphi \left(V_{w}^{*}\right){\text{PSP}}_{i}\right)\;,$$ (8)

where

$${\tilde{\text{PSP}}}_{i}\left(t\right)=\frac{1}{\tau }\int_{0}^{\infty }ds\;e^{-\frac{s}{\tau }}{\text{PSP}}_{i}\left(t-s\right)$$ (9)

is the low-pass filtered postsynaptic potential at synapse *i*, *φ*(*U*) and $\varphi (V_{w}^{*})$ are the instantaneous firing rates based on the somatic potential and the attenuated dendritic potential, respectively, and *η* is the learning rate. The factor of potentiation *α* that scales the potentiation term is positive but smaller than the inverse of the largest nudging factor 1/max_*t*_ *λ*(*t*) to prevent the unbounded growth of synaptic strengths.

Under the assumption of a periodic environment, rich dendritic input dynamics, constant nudging factor *λ* and linear *φ* (Methods), the weight dynamics in Eq 8 leads to prospective coding by making the dendritic rate $\varphi (V_{w}^{*}\left(t\right))$ approach the expected future discounted somatic input rate, i.e.

$$\varphi \left(V_{w}^{*}\left(t\right)\right)=\frac{\alpha }{\tau }\int_{0}^{\infty }ds\;e^{-\frac{s}{\tau_{\text{eff}}}}\varphi \left(U^{*}\left(t+s\right)\right),$$ (10)

where the effective discount time constant *τ*_eff_ is given by

$$\tau_{\text{eff}}=\frac{\tau }{1-\lambda \alpha }\;.$$ (11)

Depending on the factor of potentiation *α* and the nudging factor *λ*, the effective time constant *τ*_eff_ can be much larger than the biophysical time constant *τ* of low-pass filtering and match behavioral timescales of seconds. In particular, if the somatic input is strong and hence *λ* close to 0 (close to ‘clamping’), the effective discount time constant is short, *τ*_eff_ ≈ *τ*. But when nudging is weak (*λ* close to 1), the synapses on the dendrite learn to predict their self-generated somatic firing rate and the effective discount time constant is extended up to $\tau_{\text{eff}}\approx \frac{\tau }{1-\alpha }$. The case of weak nudging is also the case when the neuron’s somatic firing rate is roughly determined by the dendritic input, $\varphi \left(U\left(t\right)\right)\approx \varphi \left(V_{w}^{*}\left(t\right)\right)$, see Eq 4. In particular, if after learning the somatic input is transiently silenced, the neuron’s firing rate *φ*(*U*(*t*)), according to Eq 10, represents the discounted future rate of the somatic input U*(*t*) applied during the previous learning period, even if this was only slightly nudging the somatic potential *U*(*t*) itself.

Periodic inputs are unrealistic in a natural setting. But a similar result holds also in more general settings, where a neuron is occasionally exposed to correlated dendritic and somatic inputs. In this more general stochastic setting we derive the main result under the assumption that dendritic and somatic inputs depend on the state of a stationary latent Markov chain *X*_0_,*X*_1_, …. The dependence on a stationary latent Markov chain assures that the neuron is occasionally exposed to correlated dendritic and somatic inputs. The main result in this setting is (cf. Eq 48)

$$\varphi \left(V_{w}^{*}\left(x\right)\right)=\frac{\alpha }{1-\lambda \alpha }\sum_{k=0}^{\infty }\gamma_{\text{eff}}^{k}E\left[\varphi \left(U^{*}\left(X_{k}\right)\right)|X_{0}=x\right]\;,$$ (12)

where $\gamma_{\text{eff}}=e^{-\frac{\delta }{\tau_{\text{eff}}}}$ is a large discount factor that leads to a similar discount behavior as in the time-continuous case, if *t* = *kδ*.

It is important to note that in the stochastic case the dendritic rate is only informative about *expected* future somatic inputs. Metaphorically speaking, a neuron can learn to predict the expected win in a lottery, but obviously it cannot learn to predict single lottery draws.

### The bootstrapping effect of predicting the own predictions

**Fig 2:**
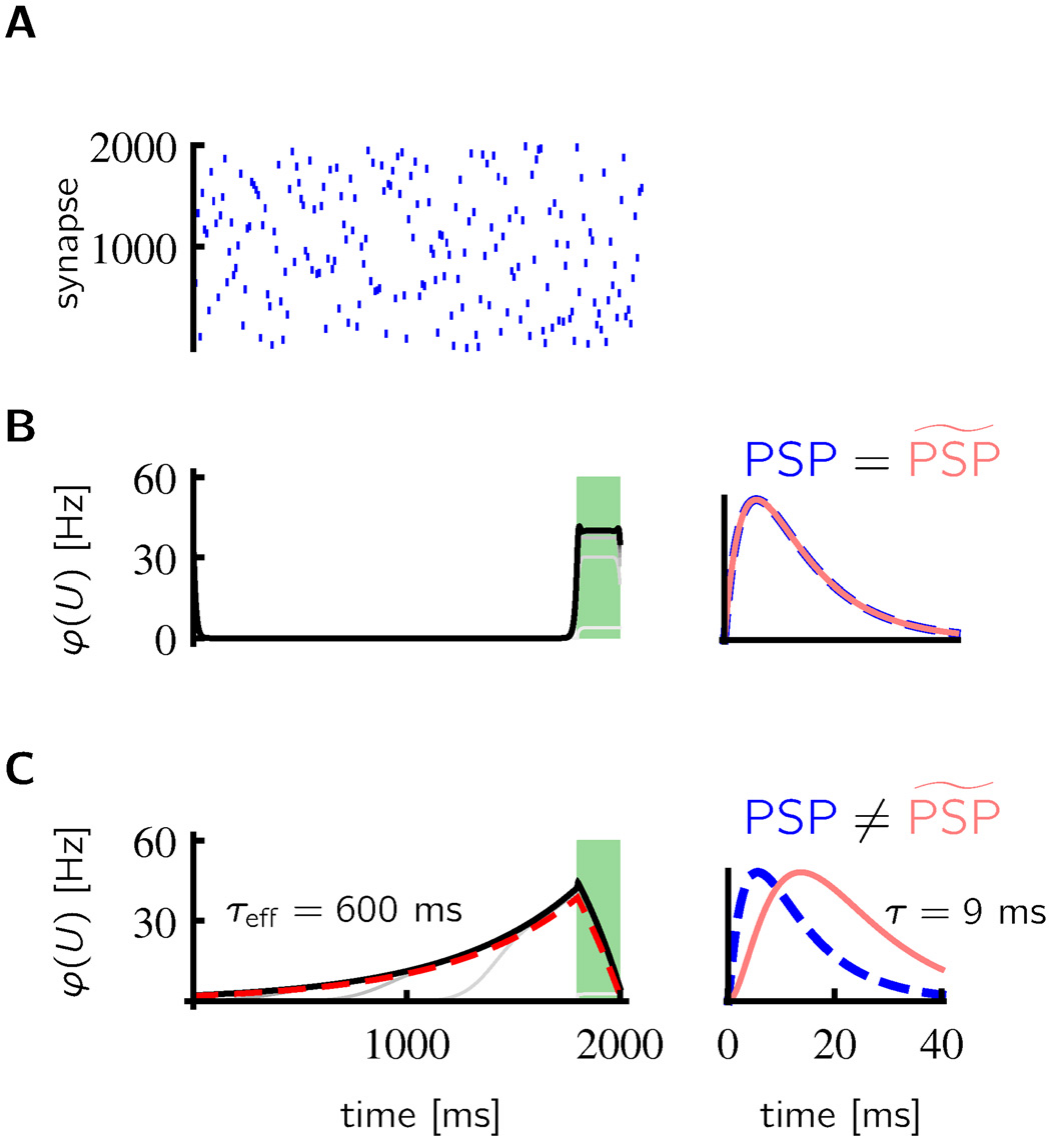
Learning activity ramps on a behavioral timescale with a biologically plausible plasticity window. **A**-**B** For orthogonal input patterns (exactly one presynaptic spike arrives at each synapse during 2 s) (A) and a somatic input after 1800 ms (*g*_*E*_ = 15 nS during green shading in B and C, *g*_*E*_ = 0 otherwise), the learned postsynaptic firing rate has a similar time course as the somatic input if $\text{PSP}=\tilde{\text{PSP}}$ (B, lines from light gray to black: postsynaptic firing rate after 100, …, 1000 training sessions). **C** If $\text{PSP}\neq \tilde{\text{PSP}}$ (with *τ* = 9 ms), the learned postsynaptic firing rate ramps up with an effective time constant of *τ*_*eff*_ = 600 ms towards the onset of the somatic input. The theoretical result is in good agreement with the simulation (dashed red line: $\varphi (V_{w}^{*})$ computed by Eq 10). During training, the 2 s long pattern of dendritic and the somatic inputs is periodically repeated.

In the limit, *τ* → 0 we find that $\tilde{\text{PSP}}=\text{PSP}$ and with *α* = 1 we recover the learning rule of Urbanczik & Senn [18]. This rule adapts the dendritic synapses such that the dendritic input matches the somatic input Fig 2B. On the other hand, the learning rule with a slightly larger potentiation window leads to dendritic input that ramps up long before the onset of somatic input Fig 2C.

By looking at Eqs 4 and 8 we can now obtain a better intuition for the bootstrapping effect of predicting the own predictions. If at the beginning of learning all synaptic weights *w*_*i*_ are zero, the dendritic potential *V*_*w*_ is at rest (= 0) all the time and the somatic membrane potential *U*(*t*) follows the somatic input U*(*t*) (see Eq 4). In this case, the learning rule in Eq 8 contains only the potentiation term

$$\dot{w}=\eta \;\alpha \;\varphi \left(U^{*}\right){\tilde{\text{PSP}}}_{i}\;.$$ (13)

In the example in Fig 2C, the somatic input U* and consequently *φ*(U*) is non-zero only after 1800 ms. Therefore, synapse *i* is potentiated only if a presynaptic spike arrives shortly before the onset of the somatic input. The next time a presynaptic spike arrives at synapse *i*, the somatic membrane potential is depolarized by the dendritic input already before the onset of the somatic input and the learning rule contains at this moment (e.g. at 1780 ms in Fig 2C) the terms

$$\dot{w}=\eta \left(\alpha \;\varphi \left(\lambda V_{w}^{*}\right){\tilde{\text{PSP}}}_{i}-\varphi \left(V_{w}^{*}\right){\text{PSP}}_{i}\right)\;.$$ (14)

These terms would cancel each other in the case of Urbanczik & Senn [18] where *α* = *λ* = 1 and ${\tilde{\text{PSP}}}_{i}={\text{PSP}}_{i}$. But if ${\tilde{\text{PSP}}}_{i}$ is the low-pass filtered version of the postsynaptic potential (as in Fig 2C) they do not cancel. Instead, synapses are potentiated, if a presynaptic spike arrives shortly before the somatic potential was depolarized due to dendritic input through already potentiated synapses. The consequence of this bootstrapping effect appears in Fig 2C in the gray curves. After 100 training sessions, the dendritic input starts to rise around 1200 ms, but synapses with earlier presynaptic spikes are not yet strengthened. With each further training session the dendritic input rises earlier.

The dendritic and the somatic inputs are deterministic periodic functions, in the example in Fig 2C. Therefore we can directly compare the simulation to the theoretical results of the previous section. For the interval without somatic input (0–1800 ms), where $\varphi \left(U\right)=\varphi \left(V_{w}^{*}\right)$, we find a good agreement (dashed red and thick black line in Fig 2C). Small differences are to be expected, because in the theoretical derivations a constant nudging factor *λ* is assumed and the steady-state solution of the somatic membrane potential dynamics is used (see Eq 4). The dendritic rate $\varphi (V_{w}^{*})$ is only slightly below the somatic rate *φ*(*U*) in the interval with somatic input (1800–2000 ms), because the somatic input is small.

### Dependence on the dendritic input structure

**Fig 3:**
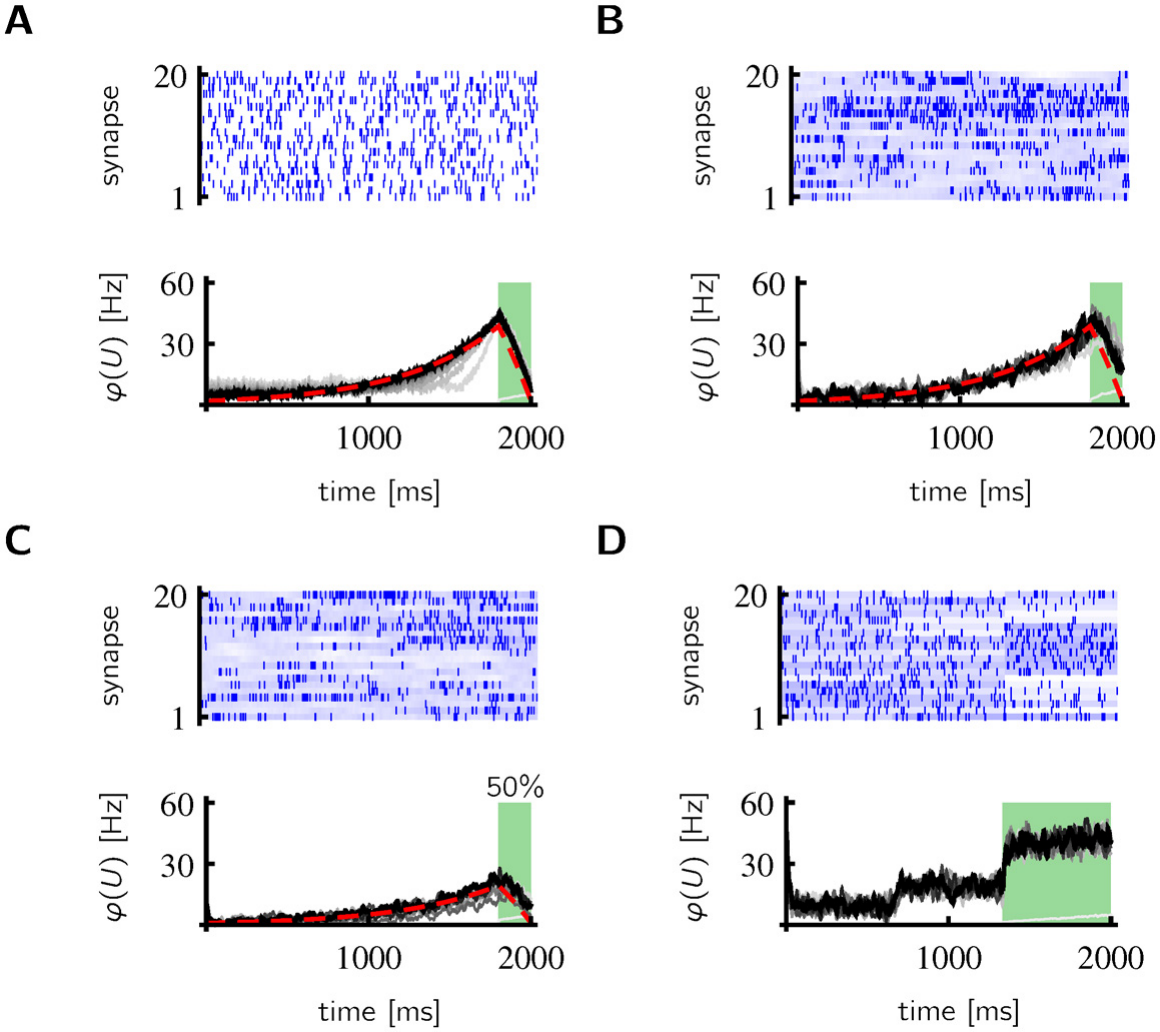
Dependence of learning on the type of input. **A** Learning succeeds with deterministically repeated spike trains (20 frozen spike trains out of 500, *g*_*E*_ as in Fig 2). **B** Learning succeeds with stochastic spiking, if the spiking rate is variable (blue shading: spiking rate, blue ticks: one sample). **C** The amplitude of the ramp is smaller, if the training event occurs only with 50% probability. **D** If the spiking rate is constant during long intervals, the input is not sufficient to learn a smooth ramp. A stepwise ramp is learned instead (*g*_*E*_ = 8 nS during green shading)

The input pattern in Fig 2A is a particularly simple example of a deterministic, periodic pattern with rich enough structure. Enough structure to learn a prospective code exists also in sufficiently many randomly generated (frozen) spike trains that are deterministically repeated, if there is always at least one presynaptic spike within the duration of a *PSP* and the probability of repeating a nearly identical presynaptic spike pattern is low (see Fig 3A). We did not systematically search for the minimal number of required dendritic synapses. But for the example in Fig 3A we found empirically that a few hundred synapses are necessary. If the presynaptic firing frequency is only 2 Hz, we found that 1000 presynaptic neurons are enough to learn the ramp in 100 trials, whenever the learning rate is larger than in the 20 Hz case. At the end of learning, the time course of the somatic potential matches the one of the previous example (black lines in Figs 2C and 3A). But during learning, the time course of the somatic potential is different in the two examples (gray lines in Figs 2C and 3A). This is a consequence of the influence of correlations in the dendritic input. For the frozen spike trains, the presynaptic auto-correlation $E[{\text{PSP}}_{i}\left(t\right){\text{PSP}}_{i}\left(t+s\right)]\neq 0$ is non-vanishing for all *s* and *i*. This causes the average firing rate to increase early during learning (Fig 3A; gray lines in interval 0–1500 ms in contrast to gray lines in the same interval in Fig 2C).

In the examples given so far, the dendritic and the somatic inputs are deterministic, but deterministic repetitions of the exact same spike trains are unrealistic. In Fig 3B we consider the more realistic case of random spiking. In each trial, the spikes are sampled from an inhomogeneous Poisson process, with periodically repeating rates. The resulting activity ramp is noisier but in good agreement with the theoretical result. It is important that the rates of the Poisson process have sufficiently rich structure. In Fig 3D the firing rate of the Poisson process is kept constant for one third of the trial. In this case, the temporal structure is not sufficiently rich to learn a smooth ramp and a stepwise activity ramp is learned instead.

In Fig 3C, the target event occurs only with a 50% chance, i.e. the somatic input is given only in half the trials. This results in an activity ramp with smaller amplitude, which is consistent with the theoretical finding that the dendritic rate depends linearly on the average somatic input rate (see Eq 12).

### Delayed paired-associate task

Prospective coding in neurons of the prefrontal cortex was observed in an experiment with monkeys performing a delayed paired-associate task [1]. In this experiment, monkeys learned to associate a visual sample to a visual target presented one second later. Our learning rule allows for learning a prospective code in such a task.

**Fig 4:**
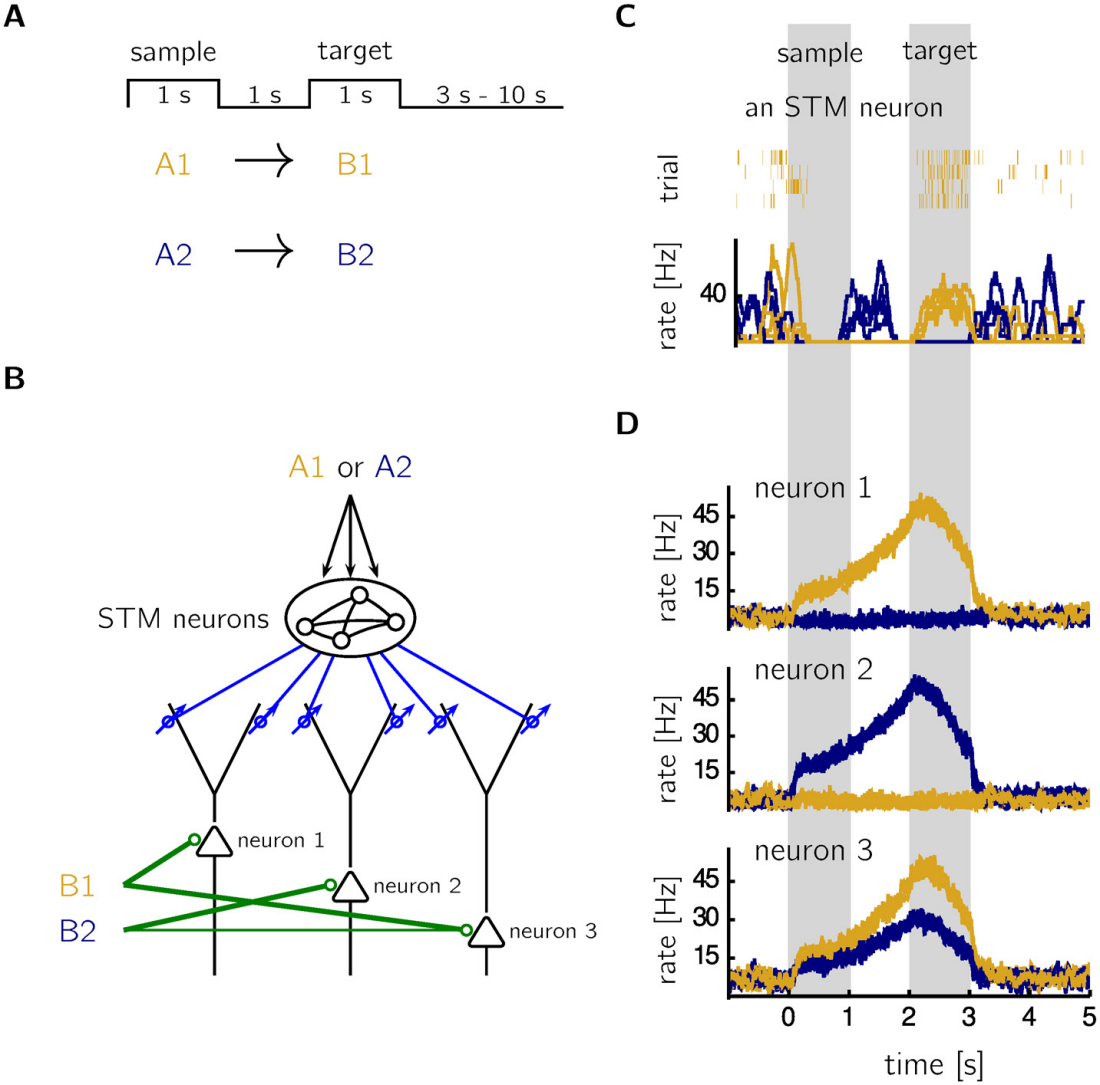
Prospective coding in a delayed paired-associate task. **A** In the simulation, stimulus A1 and A2 is repeatedly followed by stimulus B1 and B2, respectively, with a delay of 1s. The two pairs are chosen randomly with equal probabilities. Intertrial intervals are chosen at random uniformly between 3 s and 10 s. **B** The first stimulus (A1 or A2) activates a recurrent network of 2000 neurons representing a short-term memory (STM). The dynamics of the recurrent network is modeled by a stochastic process (see Methods). The STM is read out by 3 neurons that encode in a distributed manner the second stimulus (setting *g*_*E*_ = 5 nS in neuron 1 and neuron 3 during the B1 presentation, and *g*_*E*_ = 5 nS in neuron 2 and *g*_*E*_ = 2.5 nS in neuron 3 during the B2 presentation). **C** The time course of the firing rates of neurons in the recurrent short-term memory network depends on the first stimulus (dark blue: A1; gold: A2; spike trains of a specific STM neuron during 4 A1 trials and its estimated rates for 4 A1 and 4 A2 trials). **D** After learning, the firing rate of neuron 1 ramps up after stimulus A1 (gold trace), but not after stimulus A2 (blue trace). The opposite holds for neuron 2. Since neuron 3 receives more somatic input when B1 is present, the firing rate of neuron 3 ramps up to a larger value after A1 than after A2.

During training, sample A1 is always followed by target B1 after a delay of 1s, and sample A2 is followed by target B2 (Fig 4A). In the simulation we assume that the sample (first stimulus) leaves a memory trace in form of a spatio-temporal activity pattern that projects through dendritic synapses, while the target (second stimulus) drives somatic synapses (Fig 4B). In order to have sufficiently rich presynaptic activity (c.f. Fig 3B), the memory trace of the sample is modeled by an inhomogeneous Poisson process with sample dependent rate trajectories (Fig 4C), i.e. during the presentation of the first stimulus the rate trajectory of each neuron approaches a previously chosen template trajectory that depends on the sample (see Methods). These memory traces are inspired by liquid state machines (see Discussion). If a neuron receives strong somatic input only in the presence of a specific target (neurons 1 and 2 in Fig 4B), its firing rate ramps up exclusively in anticipation of this target (neurons 1 and 2 in Fig 4D). In contrast to such a ‘grandmother-cell coding’ (one neuron for one target), a set of neurons could encode the target in a distributed manner, where the target is identified by the overall activity pattern and single neurons respond differently to different target stimuli. Such a distributed code can be learned with neurons that receive somatic input of target-specific strengths (neuron 3 in Fig 4B; B1 stronger than B2). After learning, the amplitude of the activity ramp reflects this target specificity (neuron 3 in Fig 4D).

### Prospective coding of times series

**Fig 5:**
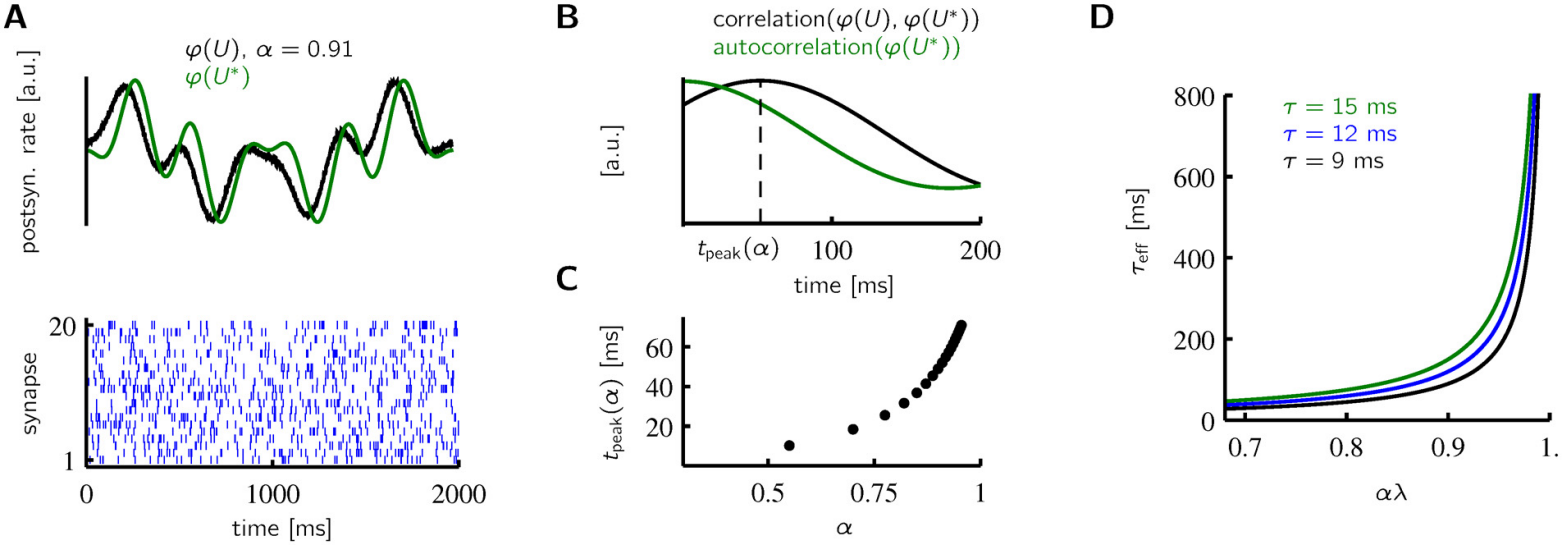
Prospective coding of time-varying input. **A** Learning leads to an advancement of the postsynaptic firing rate. The dendritic input consists of spike trains of 2000 neurons (bottom; 20 shown). The somatic input is given by *g*_*E*_(*t*) = 6(1 − sin(*ωt*) sin(2*ωt*) cos(4*ωt*)) nS, with *ω* = 2*π*/(2000 ms). **B** The correlation of the firing rate curves in A peaks at *t*_peak_ = 52 ms. **C** The advancement increases with the potentiation factor *α*. **D** With increasing potentiation factor *αλ* the effective discount time constant *τ*_eff_ becomes much larger than *τ* (Eq 11).

In Figs 2 to 4 the somatic target input was silent most of the time and active only during a short interval. This simple time course of the somatic input is, however, not a requirement and learning also converges for more complex trajectories of somatic input. In general, a time varying input through (static) somatic synapses induces plasticity that advances the postsynaptic firing rate *φ*(*U*(*t*)) relative to the firing rate *φ*(U*(*t*)) determined by the somatic input alone. Fig 5A shows an example with an advancement of roughly 50ms that has been achieved with a shorter time window (∼20 ms) for synaptic potentiation. As in Fig 3A, the dendritic input was a periodically repeated random spike train that could also be replaced by stochastic spiking with time dependent firing rates as in Fig 3B.

Since the learning rule converges to a point where the dendritic input is proportional to the future discounted somatic input (Eq 10), the advanced sequence (black in Fig 5A) is not simply a forward shifted version of the somatic input (green in Fig 5A). This becomes clearly apparent at the center of the figure, where the somatic input is symmetric around 1000 ms, but the advanced sequence is decaying, because the somatic input has a strong dip around 1100 ms. Despite this, the advancement can be characterized by the peak time of the correlation function between *φ*(*U*(*t*)) and *φ*(U*(*t*)) that, as the effective discount time constant *τ*_eff_, diverges with increasing potentiation factor *α* (Fig 5B–5D).

Time series prediction is a fundamental operation of the brain that is, for instance, involved in motor planning. In our context, the activity time course that has to be reproduced may be provided by proprioceptive feedback from muscles as somatic input U* to neurons in the primary motor cortex [19]. This feedback can be weak, delayed and sparse. The dendritic input V*, in turn, may be conveyed by a higher visual area or a premotor planning area. This dendritic input learns to predict the discounted future firing rate caused by the somatic input, and hence learns to produce the muscle activity that feeds back again as a delayed proprioceptive signal.

### Prospective coding in a recurrent neural network

**Fig 6:**
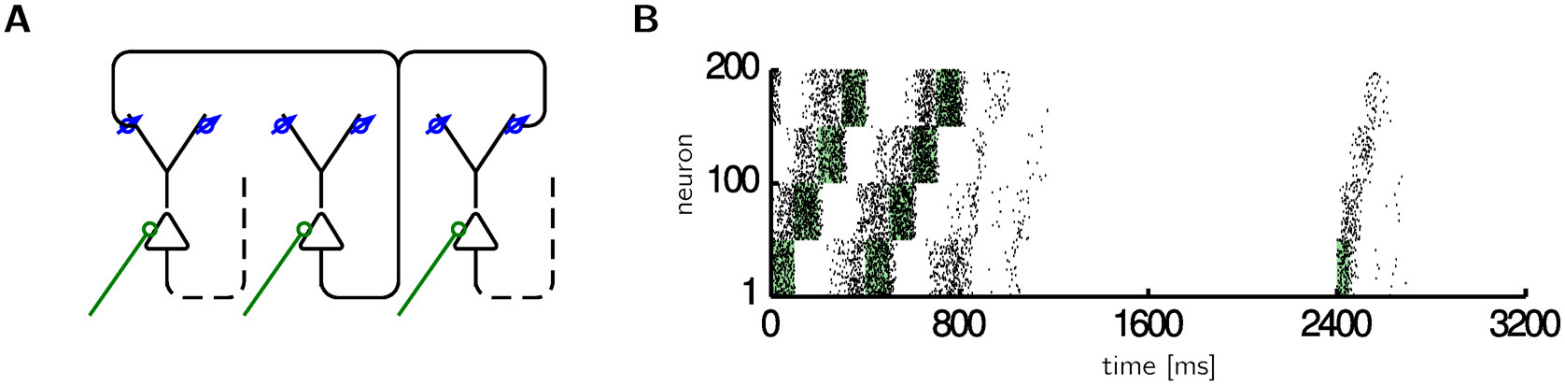
Prospective coding in a recurrent neural network. **A** The dendritic input consists of the spike trains of other neurons within the same network (for clarity only one axon is completely drawn; in the simulation we used all to all connections). **B** Groups of 50 neurons receive sequential somatic input (*g*_*E*_ = 20 nS during green shading) of duration 100 ms. After repeatedly stimulating, the firing rate increases already prior to somatic input (see for example neurons 51–100 in the first 100 ms). Before 800 ms the last two of 300 training repetitions are shown. Afterwards, no somatic input is provided anymore except for a brief stimulation (after 2400 ms), after which the sequence is autonomously replayed at a faster speed.

Lastly, we consider a recurrently connected network of 200 neurons that receive external input only at the soma and no external input at the dendrites. The input at the dendrites is given by the output spikes of the network neurons, where we consider all-to-all connectivity (Fig 6A). In contrast to the examples in Figs 2 to 5, there is no external control to assure the richness of the dendritic input and there are no guarantees that learning converges in the sense of Eq 10. Still, we observe the interesting result that learning changes synaptic strengths to allow fast replay of slow experienced sequences.

For sequentially and periodically repeated stimulations on a slow timescale (green shading in Fig 6), the recurrent dendritic connections between subsequently stimulated groups of neurons are strengthened. After 300 repetitions of the same sequence, a brief initial stimulation is sufficient to evoke an activity sequence that has the same ordering as the original sequence (Fig 6B after 2400 ms). However, the replay dynamics can be much faster than the dynamics of the stimulation. Replay depends on the internal dynamics of the network, notably the time constants of the *PSP* and the membrane time constant. Due to prospective coding, the sequence becomes advanced in time while repeatedly presenting the stimuli, and due to the recurrent connectivity the advanced sequence can be recalled with a brief stimulation of the first group of neurons (Fig 6B). Note that there is no need to explicitly distinguish between a training and recall session. Recall differs from training only in the somatic input, which consists of a brief activation of the first group of neurons during recall and slow, sequential activation during training. The learning rule is active all the time.

### Relation to TD(*λ*)

The proposed learning mechanism of prospective coding is related to a well studied version of temporal difference (TD) learning. Using our notation for a stochastic and time discrete setting, the goal in TD learning is to estimate a value function

$$V\left(x\right)=\frac{\alpha }{1-\alpha \lambda }\sum_{t=0}^{\infty }\gamma_{\text{TD}}^{t}\;E\left[\varphi \left(U^{*}\left(X_{t}\right)\right)|X_{0}=x\right]\;,$$ (15)

where 0 < *γ*_TD_ < 1 is a discount factor and the expectation is taken over the Markov chain *X*_0_,*X*_1_, …. We assume that this value function can be approximated by a linear function of the form

$$\hat{V}\left(x\right)=\varphi \left(V_{w}^{*}\left(x\right)\right)\;,$$ (16)

where *φ* is linear. In TD(*λ*) with linear function approximation, the weights *w* evolve according to the learning rule [20–22]

$$\Delta w_{t,i}=w_{t+1,i}-w_{t,i}=\eta \;\delta_{t}\;{\hat{\text{PSP}}}_{t,i}\;,$$ (17)

with learning rate *η*, eligibility trace ${\hat{\text{PSP}}}_{t,i}=\sum_{s=0}^{\infty }{\left(\lambda_{\text{TD}}\gamma_{\text{TD}}\right)}^{s}{\text{PSP}}_{i}\left(X_{t-s}\right)$, 0 ≤ *λ*_*TD*_ ≤ 1, and delta error

$$\delta_{t}=\frac{\alpha }{1-\alpha \lambda }\varphi \left(U^{*}\left(X_{t}\right)\right)+\gamma_{\text{TD}}\;\varphi \left(V_{w_{t}}^{*}\left(X_{t+1}\right)\right)-\varphi \left(V_{w_{t}}^{*}\left(X_{t}\right)\right)\;.$$ (18)

This delta error is zero on average if the approximation $\varphi (V_{w_{t}}^{*}\left(x\right))$ is equal to the value function V(x) in Eq 15. Furthermore, $\varphi (V_{w_{t}}^{*}\left(x\right))$ converges to V(x) under the learning rule of TD(*λ*) in Eq 17 [20]. The discrete time version of our learning rule (Eq 42), implemented in the 2-compartment model, converges to Eq 12 which is identical to the value function in Eq 15 if *γ*_TD_ = *γ*_eff_. Therefore, this form of TD(*λ*) and our learning mechanism converge to the same value. It is also interesting to see that both methods use an eligibility trace $\hat{\text{PSP}}$ and $\tilde{\text{PSP}}$ that are the same if *λ*_TD_ *γ*_TD_ = *γ*, i.e. *λ*_TD_ = *γ*/*γ*_eff_. But despite the convergence to the same point and the use of the same eligibility trace, learning moves in general along different trajectories under this form of TD(*λ*) and the learning mechanism we propose.

So far we compared the learning mechanism of prospective coding to the plain TD(*λ*) that has access to the *PSP* and U*. If only access to *U* = λV*+U* is available, it is also possible to combine TD(*λ*) with the bootstrapping effect of predicting the own predictions by implementing a variant of TD(*λ*) in the dendritic compartment of the 2-compartment model. If the delta error is defined as

$$\delta_{t}=\alpha \varphi \left(U(X_{t})\right)+\gamma \;\varphi \left(V_{w_{t}}^{*}\left(X_{t+1}\right)\right)-\varphi \left(V_{w_{t}}^{*}\left(X_{t}\right)\right)\;,$$ (19)

one can show that the learning rule in Eq 8 is almost identical to the TD learning rule in Eq 17 with *λ*_TD_ = 1 (Methods). In this case, the weights during learning move along similar trajectories, irrespective of whether this form of TD(1) or our learning rule is used. If this form of TD(1) were not implemented in the 2-compartment model, i.e. if the first term in the delta error in Eq 19 would be replaced by *φ*(U*(*X*_*t*_)), the time constant of future discounting would be *γ* instead of *γ*_eff_. But since the first term in the delta error in Eq 19 depends on the full somatic potential *U* = λV* + U* the bootstrapping effect of predicting the own predictions applies and the large time constant *γ*_eff_ arises.

## Discussion

As a simple and biologically plausible explanation for how animals can learn to predict future events, we have proposed a local plasticity mechanism that leads to prospective coding in spiking neurons, i.e. the plastic synapses change such that the neuron’s current firing rate depends on its expected, future discounted firing rate.

Our model proposes a partial solution to the problem of learning associations on a behavioral timescale without using slow intrinsic processes. Even with a plasticity window that is only slightly larger than the duration of a postsynaptic potential, the effective time constant of discounting the expected future firing rate can be on the order of seconds, thanks to the bootstrapping effect of predicting the own predictions. This effect arises because already predictive inputs influence the activity of a neuron. This is captured by the 2-compartment model of Urbanczik & Senn [18], where the output depends on both the dendritic (student) and the somatic (target) input.

For clarity, we presented the model with target input through static (i.e. non-plastic) somatic synapses and in the examples of ramping activity in Figs 2 and 3 the somatic input was non-zero only during a short period. This simple form of the target input is not a requirement. First, the learning mechanism also applies to arbitrary time courses of the somatic input, as we show in the example of time series prediction in Fig 5, where an advanced and smoothed version of a complex somatic input is learned. Second, the somatic synapses do not need to be static. Yet, they should change slower than the dendritic synapses in order to get a separation of plasticity timescales. And third, the target input could also arrive at another dendritic branch instead of the soma (see generality of the results in Methods).

We focused solely on learning temporal associations and neglected important aspects of learning in animals. However, the proposed learning mechanism can easily be extended to include, for example, a weighting based on behavioral relevance. In the delayed paired-associate task, our model learns the associations between sample and target irrespective of the behavioral relevance of this association. In animal training, however, reward or punishment is crucial; for example the monkeys in the study of Rainer et al. [1] received juice rewards. The learning rate in our learning mechanism is a free parameter that could incorporate a weighting by behavioral relevance. Biophysically, a neuromodulator like dopamine could implement this modulation of the learning rate. It is also possible to postpone the weight update in Eq 1 and use reward modulated eligibility traces instead (see e.g. [23–25] for theory and [15, 26] for experiments).

The proposed learning mechanism could also be involved in classical trace conditioning, where the first stimulus (CS) is separated from the second stimulus (US) and the response (R) by a delay period, similar to the situation in the delayed paired-associate task. Let us assume that neuron 1 in Fig 4 is involved in initiating response R (e.g. salivation). If the unconditioned stimulus causes somatic input to this neuron and a memory trace of the conditioned stimulus arrives at the dendritic synapses, our learning mechanism would lead to ramping activity and salivation prior to the onset of the unconditioned stimulus that originally triggered the salivation. To our knowledge, there is no conclusive experimental data to support or discard the hypothesis that prospective coding is involved in classical trace conditioning. In the cited studies on ramping activity [1, 2, 6–11], the animals were actively engaged in a task (operant conditioning). It is unlikely, however, that the ramping activity is merely a side-effect of movement preparation, since Rainer et al. [1] found it to be stimulus-specific but not action-specific.

In our model of delayed paired-associate tasks, activity ramps rely on temporally structured input from short-term memory neurons. The usage of these short-term memory neurons is motivated by the observation that hippocampal activity is needed to overcome the temporal discontiguity in trace conditioning [4, 27]. We modeled the dynamics of the recurrent short-term memory network with a stochastic process. The parameter choice of this stochastic process is inspired by the widespread experimental observation that stimulus onset quenches the neural variability [28, 29]. It should also be possible to model the memory traces with “dynamical attractors” in recurrent networks of rate neurons [30] or with long and stable transient dynamics in balanced networks [31]. Since these memory traces are not the main focus of this study we generated them in a simpler way with the stochastic process, which still feels more natural than the delay-line like traces used in a study on trace conditioning [32].

In recurrent neural networks the learning rule of prospective coding allows fast replay of slow input sequences (Fig 6). Fast replay could be valuable for planning, where it is important to quickly assess the likely successors of a given state. The same fast replay of a previously induced slower activity sequence was also observed in the rat primary visual cortex [33] and it is as well studied as compressed hippocampal replay of a spatial sequence [34]. In rats these replay events can be observed minutes or hours after the spatial experience. In contrast, the simple form of the plasticity rule in Eq 8 does not have any consolidation properties and ongoing pre- and postsynaptic activity would quickly change the learned weight patterns and thus overwrite the memories. It is, however, straightforward to extend the plasticity model by a consolidation mechanism. In the three state consolidation model of Ziegler et al. [35, 36], early long-term potentiation (LTP) is induced by a triplet rule [37]. Replacing the triplet rule by the plasticity rule in Eq 8 would endow the learning rule of prospective coding with a consolidation mechanism. Such a consolidation mechanism would allow to replay sequences a long time after the training session.

Aiming at a better understanding of biological implementations of prediction learning, our model allows to speculate about physiological realizations of the model variables. Similar to previously proposed plasticity rules [16, 18], our learning mechanism depends on the postsynaptic firing rate *φ*(*U*), a function of the dendritic potential $\varphi (V_{w}^{*})$, the postsynaptic potential *PSP* and, as a new ingredient compared to previous propositions [16, 18]: a low-pass filtered version of the postsynaptic potential $\tilde{\text{PSP}}$. A plasticity window that is slightly larger than the duration of a postsynaptic potential is in agreement with experimentally measured plasticity window sizes [13, 38]. In particular, an increased level of dopamine was observed to expand the effective time window of potentiation to at least ∼45 ms [38]. Importantly, even with a plasticity window on this timescale, predictions can be learned on a timescale of seconds due to the bootstrapping effect of predicting the own predictions.

We have shown that the proposed learning mechanism is closely related to temporal difference learning with eligibility traces TD(*λ*). As discussed in the previous paragraph, a local biological implementation of our learning rule seems straightforward. In contrast, it seems more challenging to locally implement the delta error of TD learning. Potjans et al. and Kolodziejski et al. propose a local implementation that depends either on differential Hebbian plasticity [39] or on two postsynaptic activity traces with different time constants to approximate the difference in the delta error [40]. Both methods require a gating mechanism that allows plasticity only shortly after the onset of a new state and they require transition intervals between states of fixed duration. Furthermore, “state neurons” are only highly active when the agent is in a certain state, which requires the segmentation of the sensory input stream into discrete states. The learning rule we propose does not require these strong assumptions.

Frémaux et al. [41] speculate about a non-local implementation of TD learning with spiking neurons, where the TD error is represented by the firing rate of dopaminergic neurons that receive input from three groups of neurons that encode reward, value function and derivative of the value function. In the simulations, however, Frémaux et al. did not use the proposed network implementation of the TD error and they mention that it remains to be seen whether such a circuit can effectively be used to compute a useful TD error. A non-local implementation of the TD error appears compelling in a actor-critic setting, since the actor and the critic can be learned with the same TD signal. However, if the task is to predict more than a scalar quantity like reward, it seems inefficient to use a non-local implementation of the TD error for each quantity to be predicted. Already in our simple example of prospective coding in a recurrent neural network, four TD error networks would be needed in such a non-local implementation.

Generally, associating temporally separated events requires some memory of the first event until the second event is present. Possible neural implementations of this memory rely on long spiking activity traces or on long synaptic eligibility traces. Our model of the delayed paired-associate task relies on long spiking activity traces. The short-term memory network can be seen as a liquid state machine [42] or echo state machine [43] and the ramping activity is learned as readout from this activity traces. Alternatively, the activity trace could be represented by slowly, exponentially decaying spiking activity after strong stimulation of a cell [44]. This proposition, however, fails to explain the experimentally observed activity ramps prior to predictable events [1, 2, 6–11]

The origin of the ramping activity observed in experiments is not yet fully understood. An alternative to our proposition can be found in recurrent neural network dynamics, where slowly ramping or decaying activity arises with appropriately tuned synaptic weights [2, 25]. In a reinforcement learning setting the time constant of the ramp can be learned by adjusting the recurrent weights with reward modulated Hebbian plasticity [45]. Data analysis of recordings in the macaque lateral intraparietal area revealed yet another candidate explanation: single neuron activity profiles could follow a step-like time course, while the averaged activity is a ramp, if the steps occur at different points in time [46].

Despite the formal link of our prospective coding algorithm to TD learning, the learning we consider is purely supervised on the level of the neuron. Yet, the same learning rule can also be used to explain conditioning experiments. Instead of the multiplicative modulation by a global reward signal, the reward signal could directly nudge the somatic compartment of the neurons and act as a teaching signal. But the learning rule would also allow for combining the somatic nudging signal with an additional modulatory factor, and nudging and modulatory signals could even be sparse and interleaved. For instance, the rule may explain the simultaneous shaping of predictive motor circuitries by sensory feedback and reward [5]. Fluctuating somatic inputs may cause behavioral variations and feedback signals may gate dendritic plasticity such that only rewarded fluctuations act as a target signal for prospective coding. It is also possible to adapt the somatic input connections directly with reinforcement learning, and a ramping activity could arise from learning a prospective code with stimulus-dependent dendritic input.

Since reward is an intrinsic component in animal training, we acquired an advanced knowledge about the neuronal bases of reward prediction. But predictions are not restricted to reward, and predicting the identity of stimuli yields more versatile information. We speculate that prospective coding is more abundant than previously thought and, as we showed, it could easily be implemented on the level of an individual neuron. This view is also consistent with the recently observed future-predicting encoding in the retina [47]. To this end, a potentiation window slightly larger than a PSP, together with the bootstrapping effect of predicting the own predictions, is a parsimonious mechanism for learning prospective codes by neurons. A characterisitics of these neurons is that their current firing rate matches their own expected future discounted rate.

## Methods

### Parameters of the neuron model

The spike response kernel *κ* in Eq 2 is given by

$$\kappa \left(t\right)=c\;H\left(t\right)\left(e^{-t/\tau_{m}}-e^{-t/\tau_{s}}\right)\;,$$ (20)

with Heaviside function *H*(*t*) = 0 if *t* < 0 and *H*(*t*) = 1 otherwise, *τ*_*m*_ = 10 ms, *τ*_*s*_ = 10/3 ms and $c^{-1}=\int_{-\infty }^{\infty }dt\;H\left(t\right)\left(e^{-t/\tau_{m}}-e^{-t/\tau_{s}}\right)$.

We set the somatic capacitance *C* = 1 nF, the leak conductance *g*_*L*_ = 100 nS, the coupling conductance *g*_*D*_ = 1.8 *μS*, and the excitatory and inhibitory reversal potential *E*_*E*_ = 14/3 and *E*_*I*_ = -1/3, respectively. The description of the excitatory conductance *g*_*E*_(*t*) is given in the figure captions. The inhibitory nudging conductance *g*_*I*_(*t*) was equal to 0 except for simulations with $\tilde{\text{PSP}}=\text{PSP}$ in Fig 2, where *g*_*I*_(*t*) = 4*g*_*E*_(*t*). The resting potential is 0 for both, the dendritic potential *V*_*w*_ and the somatic potential *U*. If one takes our unitless resting potential of 0 to correspond to -70 mV, and a potential of 1 to correspond to -55 mV, the above choices for *E*_*E*_ and *E*_*I*_ correspond to reversal potentials of 0 mV (excitation) and -75 mV (inhibition).

The instantaneous firing rate of the neuron is assumed to depend on the somatic membrane potential through function *φ*(*U*), which in the simulations has the form

$$\varphi \left(U\right)=\left\{\begin{array}{cc} \varphi_{\text{max}} & \;\text{if}\;U>1\\ 0 & \;\text{if}\;U<0\\ \varphi_{\text{max}}\cdot U & \text{otherwise,} \end{array}\right.$$ (21)

with *φ*_max_ = 0.06 kHz. In simulations with spiking, the firing rate multiplied by the simulation time step serves as instantaneous rate of an inhomogeneous Bernoulli process.

### Steady state solution of the somatic potential dynamics

For slowly enough changing *I*_tot_(*t*) and *g*_tot_(*t*), *U*(*t*) is well approximated by *I*_tot_(*t*)/*g*_tot_(*t*). To see this, we use the ansatz *U*(*t*) = *I*_tot_(*t*)/*g*_tot_(*t*) + *ϵ*(*t*) in Eq 1 and find

$$\dot{\epsilon }=-\frac{g_{\text{tot}}}{C}\epsilon -\frac{{\dot{I}}_{\text{tot}}}{g_{\text{tot}}}+\frac{{\dot{g}}_{\text{tot}}I_{\text{tot}}}{g_{\text{tot}}^{2}}\;,$$ (22)

which leads to the conclusion that the error *ϵ* is small if $|\frac{{\dot{I}}_{\text{tot}}}{g_{\text{tot}}}+\frac{{\dot{g}}_{\text{tot}}I_{\text{tot}}}{g_{\text{tot}}^{2}}|\ll 1$ during at least an interval of approximate duration $\frac{C}{g_{\text{tot}}}$.

Under these assumptions we write

$$U\left(t\right)\approx \frac{I_{\text{tot}}\left(t\right)}{g_{\text{tot}}\left(t\right)}=\lambda \left(t\right)V_{w}^{*}\left(t\right)+U^{*}\left(t\right)\;,$$ (23)

where we introduce the ‘nudging’ factor $\lambda \left(t\right)=\frac{g_{L}+g_{D}}{g_{\text{tot}}\left(t\right)}$, the attenuated dendritic potential $V_{w}^{*}\left(t\right)=\frac{g_{D}}{g_{L}+g_{D}}V_{w}\left(t\right)$, and the attenuated somatic input $U^{*}\left(t\right)=\frac{g_{E}\left(t\right)E_{E}+g_{I}\left(t\right)E_{I}}{g_{\text{tot}}\left(t\right)}$.

### Generality of the results

Our main results are robust to variations of the model. For example, the target input *I* could be given by the input through static synapses on another dendritic branch instead of synapses at the soma, i.e. *I*(*t*) = *g*_*D*_(*V*_*s*_(*t*) − *U*). In this case, the nudging factor becomes $\lambda =\frac{g_{L}+g_{D}}{g_{L}+2g_{D}}$ and is constant in time.

Modifying the depression term of the learning rule has an effect on the effective time scale *τ*_eff_, but large effective time constants are achievable in any case. If the depression term in Eq 8 would be replaced by $-\varphi \left(\lambda V_{w}^{*}\right){\text{PSP}}_{i}$, the effective time constant would be *τ*_eff_ ≈ *τ*/(1 − *α*), i.e. *τ*_eff_ would be independent of *λ* but still diverge when *α* → 1. Similarly, for a depression term given by −*φ*(*V*_*w*_)PSP_*i*_, the effective time constant would be *τ*_eff_ ≈ *τ*/(1 − *λ*_2_ *α*), with *λ*_2_ = *g*_*D*_/*g*_tot_.

In the current writing of the learning rule, Eq 8, the postsynaptic term arises as instantaneous firing rate *φ*(*U*). But this rate could also be replaced by a postsynaptic sample spike train *S*(*t*) that averages out to this same rate, 〈*S*(*t*)〉 = *φ*(*U*(*t*)). Since learning becomes slower by this sampling, we run our simulations in the form of Eq 8.

### Dynamics of short-term memory neurons in the delayed paired associate task

For each STM neuron *i* we first choose template rate trajectories $r_{i}^{1}\left(t\right)$ for stimulus A1 and $r_{i}^{2}\left(t\right)$ for stimulus A2 by sampling from a mean-zero Ornstein-Uhlenbeck process

$$dr_{i}^{s}\left(t\right)=-\theta_{1}r_{i}^{s}\left(t\right)dt+\sigma_{1}dW\left(t\right)\;,$$ (24)

where *W* is a Wiener process, 1/*θ*_1_ = 1000 ms, *σ*_1_ = 1 and *s* ∈ {1, 2}. Actual rate trajectories *r*_*i*_(*t*) were sampled from a process with trial dependent mean and time dependent variance, i.e.

$$dr_{i}\left(t\right)=-\theta_{2}\left(r_{i}\left(t\right)-\mu^{s}\left(t\right)\right)dt+\sigma \left(t\right)dW\left(t\right)\;,$$ (25)

where 1/*θ*_2_ = 100 ms,

$$\mu^{s}\left(t\right)=\left(1-\sigma \left(t\right)\right)r_{i}^{s}\left(t\right)-\sigma \left(t\right)/2$$ (26)

and *σ*^2^(*t*) = 1 if *t* < 0 s or *t* > 3 s, *σ*^2^(*t*) = 0.1 otherwise. This assures that in each trial the rate trajectories approach the template trajectories during the presentation of the sample. In between trials, the rate trajectories are independent of the template trajectories. Spike times are determined by sampling from an inhomogeneous Bernoulli process with rate *φ*(*r*_*i*_(*t*))Δ*t*, where Δ*t* is the simulation time step.

### Simulation details

The differential equations were integrated with the Euler forward method with step size 0.1 ms. We choose the learning rate *η* = 0.5 in all simulations except for the simulation in Fig 2B and 2C where *η* = 50, since the presynaptic firing rate is low. All simulations are written in C. The plots are generated with Mathematica. The source code is publicly available at https://github.com/jbrea/prospectiveCoding.

### Stationary point of learning for periodic environments

We assume a stationary environment and rich dendritic input dynamics, such that the dendritic inputs can potentially be predictive of the somatic input. There are different ways to model stationarity of the environment. One way is to restrict the inputs to depend on a stationary latent Markov chain. We consider this case in detail in the next section. Here, to present the main ideas in a mathematically simple form, we look at the artificial case, where stationarity enters through deterministic and periodic functions PSP_*i*_(*t*) and U*(*t*) with period *T*. Under this assumption, learning is at a stationary point when the changes of the weights in Eq 8 integrated over one period vanish, i.e.

$$0=\int_{0}^{T}dt\;{\dot{w}}_{i}\left(t\right)=\eta \int_{0}^{T}dt\;\left(\alpha \varphi \left(U(t)\right){\tilde{\text{PSP}}}_{i}\left(t\right)-\varphi \left(V_{w}^{*}\left(t\right)\right){\text{PSP}}_{i}\left(t\right)\right)\;.$$ (27)

Using the definition of $\tilde{\text{PSP}}$ in Eq 9 we find

$$0=\int_{0}^{T}dt\;\left(\alpha \varphi \left(U(t)\right)\frac{1}{\tau }\int_{0}^{\infty }ds\;e^{-\frac{s}{\tau }}{\text{PSP}}_{i}\left(t-s\right)-\varphi \left(V_{w}^{*}\left(t\right)\right){\text{PSP}}_{i}\left(t\right)\right)$$ (28)

$$=\int_{0}^{T}dt\;\left(\frac{\alpha }{\tau }\int_{0}^{\infty }ds\;e^{-\frac{s}{\tau }}\varphi \left(U(t+s)\right)-\varphi \left(V_{w}^{*}\left(t\right)\right)\right){\text{PSP}}_{i}\left(t\right)\;,$$ (29)

where Eq 29 is obtained by changing the order of integration, changing the integration variable *t* to *t* + *s* and using $\int_{-s}^{T-s}dt\;f\left(t\right)=\int_{0}^{T}dt\;f\left(t\right)$, which holds for any *T*-periodic function *f*(*t*). The puzzling transition from an integral that depends on the past values of PSP_*i*_ in Eq 28 to an integral that depends on the future values of *U* in Eq 29 is a result of the assumed stationarity of the environment, which here is expressed in the periodicity of the functions PSP_*i*_(*t*) and U*(*t*). Eq 29 holds for all synapses *i*, if

$$\varphi \left(V_{w}^{*}\left(t\right)\right)=\frac{\alpha }{\tau }\int_{0}^{\infty }ds\;e^{-\frac{s}{\tau }}\varphi \left(U(t+s)\right)\;.$$ (30)

Strictly, Eq 30 follows from Eq 29 only if the inputs PSP_*i*_(*t*) span the space of square integrable, *T*-periodic functions. In actual implementations the number of synapses is limited, but we find empirically that Eq 30 holds approximately at the stationary point if, loosely speaking, the inputs PSP_*i*_(*t*) at individual synapses are sufficiently rich and different from each other.

The right-hand side of Eq 30 also depends on the dendritic potential $V_{w}^{*}$, since the membrane potential *U* depends both on the dendritic input $V_{w}^{*}$ and the somatic input U* (see Eq 4). Assuming a linear transfer function *φ*, Eq 30 becomes

$$\varphi \left(V_{w}^{*}\left(t\right)\right)=\frac{\alpha }{\tau }\int_{0}^{\infty }ds\;e^{-\frac{s}{\tau }}\left(\lambda \varphi \left(V_{w}^{*}\left(t+s\right)\right)+\varphi \left(U^{*}\left(t+s\right)\right)\right)\;.$$ (31)

With a Fourier transform and assuming a constant nudging factor *λ* we can solve this equation for $\varphi (V_{w}^{*}\left(t\right))$.

The Fourier coefficients ${\hat{h}}_{k}$, k∈Z, of the *T*-periodic function $h\left(t\right)=\int_{0}^{\infty }ds\;e^{-\frac{s}{\tau }}f\left(t+s\right)$ are given by

$${\hat{h}}_{k}=\int_{0}^{T}dt\;e^{-i\frac{2\pi kt}{T}}\int_{0}^{\infty }ds\;e^{-\frac{s}{\tau }}f\left(t+s\right)=\int_{0}^{T}dt\;e^{-i\frac{2\pi kt}{T}}f\left(t\right)\int_{0}^{\infty }ds\;e^{i\frac{2\pi ks}{T}}e^{-\frac{s}{\tau }}$$ (32)

$$={\hat{f}}_{k}\int_{0}^{\infty }ds\;e^{s\left(i\frac{2\pi k}{T}-\frac{1}{\tau }\right)}$$ (33)

$$={\hat{f}}_{k}\;\frac{1}{\frac{1}{\tau }-i\frac{2\pi k}{T}}\;,$$ (34)

where, in the first line, we changed the order of integration, changed the variable *t* to *t* − *s* and used the periodicity of the integrand to obtain $\int_{-s}^{T-s}dt\;e^{-i\frac{2\pi kt}{T}}f\left(t\right)=\int_{0}^{T}dt\;e^{-i\frac{2\pi kt}{T}}f\left(t\right)$. In the second line we introduced the Fourier coefficients ${\hat{f}}_{k}$.

With $f\left(t\right)=\varphi (V_{w}^{*}\left(t\right))$ and *g*(*t*) = *φ*(U*(*t*)) we rewrite Eq 31

$$f\left(t\right)=\frac{\alpha }{\tau }\int_{0}^{\infty }ds\;e^{-\frac{s}{\tau }}\left(\lambda f(t+s)+g(t+s)\right)$$ (35)

and Fourier transform both sides to obtain

$${\hat{f}}_{k}=\frac{\alpha }{\tau }\left(\lambda {\hat{f}}_{k}+{\hat{g}}_{k}\right)\;\frac{1}{\frac{1}{\tau }-i\frac{2\pi k}{T}}\;.$$ (36)

Solving for ${\hat{f}}_{k}$ leads to

$${\hat{f}}_{k}=\frac{\alpha }{\tau }\;{\hat{g}}_{k}\;\frac{\frac{1}{\frac{1}{\tau }-i\frac{2\pi k}{T}}}{1-\frac{\alpha \lambda }{\tau }\frac{1}{\frac{1}{\tau }-i\frac{2\pi k}{T}}}$$ (37)

$$=\frac{\alpha }{\tau }\;{\hat{g}}_{k}\;\frac{1}{\frac{1-\alpha \lambda }{\tau }-i\frac{2\pi k}{T}}\;.$$ (38)

This equation has the same structure as Eq 34. With the inverse Fourier transform and assuming *αλ* < 1 we find Eq 10, i.e.

$$f\left(t\right)=\frac{\alpha }{\tau }\int_{0}^{\infty }ds\;e^{-\frac{s}{\tau_{\text{eff}}}}g\left(t+s\right)\;,$$ (39)

where $\tau_{\text{eff}}=\frac{\tau }{1-\alpha \lambda }$.

### Convergence of learning in stationary stochastic environments

We formalize the notion of a stationary environment by introducing a stationary latent Markov chain and restricting the dendritic input PSP_*i*_(*t*) = PSP_*i*_(*X*_*t*_) and the somatic input U*(*t*) = U*(*X*_*t*_) to depend on the state *X*_*t*_ of the Markov chain. An alternative way to formalize the notion of stationarity would be to define stationary dynamics of the dendritic inputs and define the correlation between dendritic and somatic input. As it is always possible to reformulate the stationary dendritic input dynamics and the correlation between dendritic and somatic input in terms of a stationary latent Markov chain—with potentially large state space—we stick to the description with a latent Markov chain.

Formally, for time t∈Z, states *X*_*t*_ in a finite set $X=(s_{1},s_{2},\ldots ,s_{N})$ evolve according to a stationary, irreducible Markov chain with transition probabilities *T*(*s*_*i*_,*s*_*j*_) = *Pr*(*X*_*t*+1_ = *s*_*j*_|*X*_*t*_ = *s*_*i*_) and stationary distribution *π*(*s*_*i*_) = *Pr*(*X*_*t*_ = *s*_*i*_).

Note that the case of deterministic periodic input is readily formulated in terms of a stationary latent Markov chain that cycles deterministically through the state space, e.g. *T*(*s*_*i*_, *s*_*j*_) = 1 if *j* = *i* + 1 or *j* = 1 and *i* = *N* and *T*(*s*_*i*_, *s*_*j*_) = 0 otherwise. Functions that depend only on the state of the Markov chain are thus cyclic with period *N*, e.g. PSP_*i*_(*X*_*t*_) = PSP_*i*_(*X*_*t*+*N*_).

In order to switch to matrix notation in the rest of this section, we introduce the following terms:

- Discounting operator $A=\alpha \sum_{t=0}^{\infty }\gamma^{t}T^{t}$, with transition matrix *T* and discount factor *γ* ∈ [0, 1).
- Postsynaptic potentials **b**_*i*_ = (PSP_*i*_(*s*_1_), …,PSP_*i*_(*s*_*N*_))′ for each synapse *i*.
- Matrix of postsynaptic potentials *B* = [**b**_1_ **b**_2_⋯**b**_*S*_], where *S* is the number of synapses.
- Postsynaptic firing rates **r**_*U*_ = (*φ*(*U*(*s*_1_)), …, *φ*(*U*(*s*_*N*_)))′.
- Dendritic rates ${\text{r}}_{V}=(\varphi (V_{w}^{*}(s_{1})),\ldots ,\varphi (V_{w}^{*}(s_{N})){)}^{\prime }$.
- Somatic input rates **r**_*I*_ = (*φ*(U*(*s*_1_)), …, *φ*(U*(*s*_*N*_)))′.
- Expected future discounted firing rate $F\left(x\right)=\left(Ar_{U}\right)\left(x\right)=\alpha \sum_{t=0}^{\infty }\gamma^{t}E[\varphi (U(X_{t}))|X_{0}=x]$.
- Expected low-pass filtered postsynaptic potential ${\tilde{\text{PSP}}}_{i}\left(x\right)=\sum_{t=0}^{\infty }\gamma^{t}E[\text{PSP}\left(X_{-t}\right)|X_{0}=x]$.

In the following we sketch the proof for the equivalents of Eqs 30, 12 and 11 in the Markov chain setting. We will make use of the following basic facts about conditional expectations:

$$E\left[f\left(X_{t}\right)|X_{0}=x\right]=\sum_{x_{0},x_{1},\ldots ,x_{t}}\delta \left(x,x_{0}\right)\prod_{s=1}^{t}T\left(x_{s-1},x_{s}\right)f\left(x_{t}\right)=\left(T^{t}f\right)\left(x\right),$$ (40)

$$E\left[f\left(X_{-t}\right)|X_{0}=x\right]=\frac{1}{\pi (x)}\sum_{x_{-t}}f\left(x_{-t}\right)\pi \left(x_{-t}\right)\text{Pr}\left(X_{0}=x|X_{-t}=x_{-t}\right)=\frac{1}{\pi (x)}\left(f^{\prime }\Pi T^{t}\right)\left(x\right),$$ (41)

where *t* > 0, *T*^*t*^ denotes the matrix power of *T*, Π = diag(*π*(*s*_1_),*π*(*s*_2_), …, *π*(*s*_*N*_)) is the diagonal “stationary distribution matrix”, column vector **f** = (*f*(*s*_1_),*f*(*s*_2_), …, *f*(*s*_*N*_))′ and row vector **f**′, the transposed of **f**.

#### 1. At the fixed point of learning the dendritic rate is proportional to the expected future discounted firing rate (cf. Eq 30)

In discrete time the learning rule in Eq 8 becomes

$$\Delta w_{t,i}=\eta \left(\alpha \varphi \left(U(X_{t})\right){\tilde{\text{PSP}}}_{t,i}-\varphi \left(V_{w_{t}}^{*}\left(X_{t}\right)\right){\text{PSP}}_{i}\left(X_{t}\right)\right)\;,$$ (42)

with ${\tilde{\text{PSP}}}_{t,i}=\sum_{s=0}^{\infty }\gamma^{s}{\text{PSP}}_{i}\left(X_{t-s}\right)$. While Δ*w*_*t*,*i*_ is a stochastic variable in general, we will discuss in the following only the corresponding ordinary differential equation (ODE) of the mean

$${\dot{w}}_{i}=\eta \sum_{x\in X}\pi \left(x\right)\left(\alpha \varphi \left(U\left(x\right)\right){\tilde{\text{PSP}}}_{i}\left(x\right)-\varphi \left(V_{w}^{*}\left(x\right)\right){\text{PSP}}_{i}\left(x\right)\right)\;,$$ (43)

where ${\tilde{\text{PSP}}}_{i}\left(x\right)=\sum_{t=0}^{\infty }\gamma^{t}E[\text{PSP}\left(X_{-t}\right)|X_{0}=x]$ is the expected low-pass filtered postsynaptic potential. This ODE has the same fixed point and convergence behavior as the dynamics in Eq 42 under mild assumptions [48].

As in Eqs 28 and 29, we are going to show now that we can rewrite the dynamics of the mean synaptic weight in terms of the future discounted firing rate *F*(*x*) instead of the expected low-pass filtered postsynaptic potential ${\tilde{\text{PSP}}}_{i}\left(x\right)$, i.e.

$${\dot{w}}_{i}=\eta \sum_{x}\pi \left(x\right)\left(F\left(x\right)-\varphi (V_{w}^{*}\left(x\right))\right){\text{PSP}}_{i}\left(x\right)\;.$$ (44)

This result is a consequence of the assumed stationarity of the Markov chain. It can be found by focusing on the potentiation term in the learning rule in Eq 43 and using Eq 41 and the notation introduced in the last paragraph, in particular,

$${\tilde{\text{PSP}}}_{i}\left(x\right)=\sum_{t=0}^{\infty }\gamma^{t}E\left[\text{PSP}\left(X_{-t}\right)|X_{0}=x\right]=\frac{1}{\pi (x)}\left(\sum_{t=0}^{\infty }\gamma^{t}b^{\prime }\Pi T^{t}\right)\left(x\right)=\frac{1}{\pi (x)\alpha }\left(b^{\prime }\Pi A\right)\left(x\right)\;,$$ (45)

which leads to

$$\sum_{x}\pi \left(x\right)\alpha \varphi \left(U\left(x\right)\right){\tilde{\text{PSP}}}_{i}\left(x\right)=b_{i}^{\prime }\Pi Ar_{U}=\sum_{x}\pi \left(x\right)\left(Ar_{U}\right)\left(x\right){\text{PSP}}_{i}\left(x\right)=\sum_{x}\pi \left(x\right)F\left(x\right){\text{PSP}}_{i}\left(x\right)\;.$$ (46)

Using this equality in Eq 43 leads to Eq 44.

Assuming a trivial kernel for *B*Π, i.e. *B*Π**x** = **0** ⇔ **x** = **0**, we find by looking at Eq 44 that

$$\forall i:\;{\dot{w}}_{i}=0\Leftrightarrow \forall x:\;\varphi \left(V_{w}^{*}\left(x\right)\right)=F\left(x\right)\;,$$ (47)

which is analogous to the statement in Eq 30. The assumption of a trivial kernel of *B*Π implies that the map PSP(*s*_*i*_) from the state space of the latent Markov chain to dendritic inputs is one-to-one. This assumption is analogous to the statement that the dendritic inputs PSP_*i*_(*t*) at individual synapses are sufficiently rich and different from each other (see Results after Eq 30).

#### 2. At the fixed point of learning the dendritic rate that is proportional to the expected future discounted somatic input rate, but with a longer discount time constant (cf. Eq 12)

Since the future discounted firing rate *F*(*x*) depends on $\varphi (V_{w}^{*}\left(x\right))$, it is not trivial to solve Eq 47 for $\varphi (V_{w}^{*}\left(x\right))$. Similar as in the result section in Eqs 31 and 10, however, we can show for a linear *φ* and constant *λ*(*s*_*i*_) = *λ* that

$$\varphi \left(V_{w}^{*}\left(x\right)\right)=\frac{\alpha }{1-\lambda \alpha }\sum_{t=0}^{\infty }\gamma_{\text{eff}}^{t}E\left[\varphi \left(U^{*}\left(X_{t}\right)\right)|X_{0}=x\right]\;,$$ (48)

with $\gamma_{\text{eff}}=\frac{\gamma }{1-\lambda \alpha }$. Indeed, assuming linear *φ* we can rewrite Eq 47 in vector notation and solve for **r**_*V*_ to obtain

$$r_{V}=Ar_{U}=A\left(\lambda r_{V}+r_{I}\right)$$ (49)

$$\Rightarrow r_{V}={\left(1-\lambda A\right)}^{-1}Ar_{I}=\sum_{s=0}^{\infty }\lambda^{s}A^{s+1}\;r_{I}\;,$$ (50)

where we have assumed that *λα* < 1 − *γ* such that the series converges. Powers of *A* evaluate to

$$A^{s+1}=\alpha^{s+1}\overset{\infty }{\underset{t_{1}=0,\ldots ,t_{s+1}=0}{\sum }}\gamma^{t_{1}+\cdots +t_{s+1}}T^{t_{1}+\cdots +t_{s+1}}=\alpha^{s+1}\overset{\infty }{\underset{t=0}{\sum }}\left(\begin{array}{c} t+s\\ t \end{array}\right)\gamma^{t}T^{t},$$ (51)

and thus we can rewrite Eq 50 to get,

$$\begin{array}{ll} r_{V} & =\alpha \overset{\infty }{\underset{t=0}{\sum^{\text{​}}}}\underset{={\left(1-\lambda \alpha \right)}^{-t-1}}{\underset{︸}{\overset{\infty }{\underset{s=0}{\sum^{\text{​}}}}{\left(\lambda \alpha \right)}^{s}\left(\begin{array}{c} t+s\\ t \end{array}\right)}}\gamma^{t}T^{t}r_{I}\\ & =\frac{\alpha }{1-\lambda \alpha }\overset{\infty }{\underset{t=0}{\sum^{\text{​}}}}{\left(\frac{\gamma }{1-\lambda \alpha }\right)}^{t}T^{t}r_{I}, \end{array}$$ (52)

which proves the claim in Eq 48. The effective time constant $\gamma_{\text{eff}}=\frac{\gamma }{1-\lambda \alpha }$ can be much larger than *γ*. In fact, for *α* → (1 − *γ*)/*λ* we find *γ*_eff_ → 1.

#### Remarks

1. For affine *φ*(*u*) = *a* ⋅ *u* + *c* the equivalent of Eq 50 is
  $$r_{V}={\left(1-\lambda A\right)}^{-1}A\left(r_{I}-c\right).$$ (53)
  In a first order approximation, the stationary **r**_*V*_ for a non-linear *φ* is thus a translated version of the stationary solution for a linear *φ*.
2. For input **r**_*I*_ = 0 and linear *φ* we find that at the stationary point of learning **r**_*V*_ = 0. Thus we expect that learned weights decay again, once input **r**_*I*_ is removed.

#### 3. Convergence of learning

For linear *φ*(*u*) = *ϕ* ⋅ *u* with *ϕ* > 0, we have **r**_*V*_ = *ϕB* **w** and thus the learning rule in Eq 43 can be written in vector notation as

$$\dot{w}=\eta B^{\prime }\Pi \left(A\left(\Lambda r_{V}+r_{I}\right)-r_{V}\right)=\underset{=X}{\underset{︸}{\eta \phi B^{\prime }\Pi \left(A\Lambda -1\right)B}}w+\underset{=c}{\underset{︸}{\eta B^{\prime }\Pi Ar_{I}}}\;,$$ (54)

where we introduced the diagonal ‘nudging matrix’ Λ = diag(*λ*(*s*_1_),…,*λ*(*s*_*N*_)).

With w* = −*X*^+^ **c** + (1 − *X*^+^ *X*)**w** the orthogonal projection of **w** onto W* = {**w**|*X* **w** = −**c**}, where *X*^+^ denotes the Moore-Penrose pseudoinverse of *X*, we are going to show that $L\left(w\right)=\frac{1}{2}{\left(w-w^{*}\right)}^{2}$ is a Lyapunov function of the dynamics in Eq 54. With **y** = **w** − w* and $z=\sqrt{\eta \phi }By$, the temporal evolution of *L* is given by

$$\begin{array}{ll} \dot{L} & ={\left(\nabla L\right)}^{\prime }\dot{w}\\ & =y^{\prime }(X(w^{*}+y)+c)\\ & =y^{\prime }Xy\\ & =\eta \phi y^{\prime }B^{\prime }\Pi (A\Lambda -1)By\\ & =\langle z,A\Lambda z\rangle -\langle z,z\rangle \leq 0, \end{array}$$

where we defined the scalar product 〈**x**,**y**〉 = **x**′ Π **y** and the inequality follows since both *A* and Λ are contracting maps, i.e. $\sqrt{\langle Az,Az\rangle }=∥Az∥\leq ∥z∥$ and therefore

〈z,AΛz〉≤∥z∥∥AΛz∥≤∥z∥∥Λz∥≤∥z∥∥z∥=〈z,z〉. (55)

Λ is contracting because it is diagonal with entries between 0 and 1 and *A* is contracting because

$${∥Az∥}^{2}=\sum_{i}\pi \left(i\right){\left(\sum_{j}A_{ij}z_{j}\right)}^{2}\leq \sum_{i}\pi \left(i\right)\underset{<1}{\underset{︸}{\left(\sum_{j}A_{ij}^{2}\right)}}\left(\sum_{j}z_{j}^{2}\right)\leq \sum_{i}\pi \left(i\right)\sum_{j}z_{j}^{2}={∥z∥}^{2}\;,$$ (56)

where we used the facts that 0 ≤ *A*_*ij*_ < 1 and the row sums of *A* are equal to $\frac{\alpha }{1-\gamma }$ and therefore $\sum_{j}A_{ij}^{2}\leq \sum_{j}A_{ij}<1$.

### Relation between the learning rule in Eq 42 and TD(1)

For *λ*_TD_ = 1 and therefore $\hat{\text{PSP}}=\tilde{\text{PSP}}$, we can rewrite Eq 17 by expanding the delta error in Eq 19 and using the identity $\gamma \;{\tilde{\text{PSP}}}_{t,i}={\tilde{\text{PSP}}}_{t+1,i}-{\text{PSP}}_{i}\left(X_{t+1}\right)$ to find

$$\begin{array}{ll} \delta_{t}{\tilde{\text{PSP}}}_{t,i} & =\alpha \varphi \left(U(X_{t})\right){\tilde{\text{PSP}}}_{t,i}+\varphi \left(V_{w_{t}}^{*}(X_{t+1})\right)\left({\tilde{\text{PSP}}}_{t+1,i}-\text{PS}{\text{P}}_{i}(X_{t+1})\right)-\varphi \left(V_{w_{t}}^{*}(X_{t})\right){\tilde{\text{PSP}}}_{t,i}\\ & =\alpha \varphi \left(U(X_{t})\right){\tilde{\text{PSP}}}_{t,i}-\varphi \left(V_{w_{t}}^{*}(X_{t+1})\right)\text{PS}{\text{P}}_{i}(X_{t+1}) \end{array}$$ (57)

$$+\varphi \left(V_{w_{t}}^{*}\left(X_{t+1}\right)\right){\tilde{\text{PSP}}}_{t+1,i}-\varphi \left(V_{w_{t}}^{*}\left(X_{t}\right)\right)\;{\tilde{\text{PSP}}}_{t,i}\;.$$ (58)

With small parameter updates in each time step, the terms in Eq 58 approximately cancel each other when summing over subsequent terms: $\delta_{t}\;{\tilde{\text{PSP}}}_{t,i}$ contributes $+\varphi (V_{w_{t}}^{*}\left(X_{t+1}\right)){\tilde{\text{PSP}}}_{t+1,i}$ and $\delta_{t+1}\;{\tilde{\text{PSP}}}_{t+1,i}$ contributes $-\varphi (V_{w_{t+1}}^{*}\left(X_{t+1}\right)){\tilde{\text{PSP}}}_{t+1,i}$. What remains are the terms in Eq 57, which resemble the terms in the learning rule in Eq 42.
